# Editorial: Horizons in integrative neuroscience 2022

**DOI:** 10.3389/fnint.2023.1290824

**Published:** 2023-10-10

**Authors:** Elizabeth B. Torres

**Affiliations:** Psychology, Computer Science, Rutgers University Center for Cognitive Science, The State University of New Jersey, New Brunswick, NJ, United States

**Keywords:** perception, motor learning, artificial neural networks, anesthesia, fNIRS, event-related potentials (ERP), fragile X

A review of motor learning theoretical ideas and empirical assays is featured in “Bridging event-related potentials with behavioral studies in motor learning” by Deng et al.. In this paper, the authors review the motor control field and differentiate between two distinct processes featuring implicit and explicit motor learning, while also distinguishing two kinds of errors that drive motor learning: sensory prediction error and task error. They highlight how separately, electrophysiologists have described two motor-related, event-related potentials (ERPs): error-related negativity (ERN), and feedback-related negativity (FRN) that nevertheless remain disconnected from the types of learning errors described in the motor control literature. This important review aims at bridging these disparate fields by bringing awareness about the research of the two groups and encouraging collaborative links of these seemingly related learning phenomena.

In line with the need to bridge motor behavior with cognitive phenomena, the review entitled “The brain in motion–cognitive effects of simultaneous motor activity” by Schmidt-Kassow and Kaiser addresses heterogeneous and often contradictory results in the literature that employs a dual behavioral task to probe their (often deleterious) effects on cognitive performance. They further highlight new advances in electroencephalography (EEG) and invite systematic investigation of a proposed hypothesis, namely, the need to find for each cognitive function, the motor activity that matches this function in terms of attentional focus. The proposal aims at reconciling disparate results from 30 years of research in the field.

Further aiming at improvements toward eliminating biases in neuroscience research, the paper entitled “Interdisciplinary views of fNIRS: Current advancements, equity challenges, and an agenda for future needs of a diverse fNIRS research community” by Doherty et al. reviews Functional Near-Infrared Spectroscopy (fNIRS) as a neuroimaging modality that promises to bring more naturalistic studies of brain activity in real-world environments. They address the broader use of fNIRS and the need to diversify the populations under study, offering ways to reduce bias in neuroscience research to make it more equitable and inclusive—an approach that is particularly important in the study of diseases and disorders of the nervous systems.

Along those lines and following one of the main tenets of Integrative Neuroscience, the paper entitled “From circuits to behavior: Amygdala dysfunction in fragile X syndrome” by Svalina et al. aims at shedding light on fragile X syndrome by targeting precise cellular and circuit-level underpinnings of amygdala-based disorders. In their review, the authors explain new findings related to the development of the amygdala, including the role of neuromodulation in the critical period plasticity, along with recent advances in our understanding of how synaptic and circuit-level changes in the basolateral amygdala contribute to the behavioral manifestations seen in FXS. The review adds to our understanding of this neurodevelopmental disorder but also highlights well-known consequences for normal cellular and synaptic development leading to a variety of neuropsychiatric disorders including an increased prevalence of amygdala-based disorders.

In their review “Formononetin: A Pathway to Protect Neurons”, Ma and Wang explain how Formononetin (FMN) has the potential for preventing and treating various diseases such as traumatic brain injury (TBI), spinal cord injury (SCI), ischemic stroke, cerebral ischemia-reperfusion, Alzheimer's disease, and nerve tumor, owing to its antioxidative, antihypertensive, antitumor, and anti-infective properties. Their literature review offers information about the signaling pathways of neuroprotection of FMN, suggesting it as a novel candidate for the development of drugs targeting the central nervous system.

Looping back to perception-action related research, “The cost of aiming for the best answers: Inconsistent perception” by Smeets and Brenner reviews various examples of inconsistencies in our perception of the values of various attributes that reveal how our assumptions of consistency in external phenomena may lead us to erroneous conclusions when actions lead to unexpected outcomes. The authors propose that perception is about using information gathered specifically for a question, i.e., to answer that question, rather than to form an internal representation that matches the external information. This proposition, they argue, would explain inconsistencies in perception and apparent dissociation between some perceptual judgements and related actions, as it is the case in the examples that they review.

And transitioning from perception to cognition, in the realm of artificial neural networks (ANNs), the paper entitled “Remembrance of things perceived: Adding thalamocortical function to artificial neural networks” by Loeb reviews work on bio-inspired models of purely cortical circuits that address issues of human intelligence. The author explains that despite their success in some aspects of intelligence, the current models fall short of providing parsimonious explanations of cognitive phenomena. The review then considers the addition to the ANNs of thalamocortical circuits, and its putative functions related to cortical attention. Such biologically inspired models, the author argues, can help provide new testable theories of biological cognition and AI-related phenomena developing at present outside the academic realm.

As our understanding of consciousness remains elusive across fields that study conscious phenomena, the use of anesthesia opens important questions when the brain is unconscious. In their paper entitled “Brain areas modulation in consciousness during sevoflurane anesthesia” by Lyu et al., the authors review sevoflurane, one of the most used inhaled anesthetics worldwide. The review covers 20 years of history on sevoflurane use and includes perspectives on functional magnetic resonance, electroencephalogram, and pharmacological experiments. The authors examine the interactions and neurotransmitters involved in brain circuits and brain regions during sevoflurane anesthesia, attempting to shed light on mechanisms behind human consciousness along more than one level of inquiry.

The cutting-edge work presented in this Article Research Topic highlights the diversity of research performed across the entire breath of Integrative Neuroscience and reflects on the latest advances in the theory, experiment, and methodology with applications to contemporary compelling problems of making science actionable, inclusive, and truly diverse.

## Author contributions

ET: Writing—original draft.

